# Analysis of catabolic products of L-arginine; L-ornithine and L-citrulline and the residual L-arginine using the HPLC and LC-MS

**DOI:** 10.1371/journal.pone.0346976

**Published:** 2026-04-24

**Authors:** Saranya Prashath

**Affiliations:** School of Biosciences, Division of Natural Sciences, University of Kent, United Kingdom; University of Nebraska Medical Center, UNITED STATES OF AMERICA

## Abstract

L-arginine, a semi-essential amino acid, is metabolised in the cell to generate nitric oxide (NO) and L-citrulline via the enzyme nitric oxide synthase (NOS) or urea and L-ornithine via arginase activity. L-citrulline and L-ornithine are the products of L-arginine degradation. Mouse liver epithelial (BNL CL2) and mouse embryonic fibroblast (3T3 L1) insulin-sensitive cell lines were used as model systems and cultured with 0, 400 or 800 µM L-Arg. This study focuses on the analysis of the residual concentrations of amino acids (L-Arg, L-Cit and L-Orn) in cell culture medium samples using high performance liquid chromatography that involves precolumn derivatization with o-phthaldialdehyde. In BNL CL2 cells, most of the culture supernatant has increased amount of L-Arg in comparison to the control complete DMEM addition. L-ornithine levels showed an overall increase over time, with higher concentrations observed at 72 h compared with 24 h across all samples. In 3T3 L1 cells, residual L-Arg concentration decreased in most of the cell supernatant in comparison to the control at 72 h. Noticeably, L-Arg at 0 µM and the control complete DMEM had highest amount of L-Orn among all samples. Interestingly, L-Cit was very much high in culture medium of both untreated BNL CL2 (85.96 µM) and 3T3 L1 (37.49 µM) cells at T = 0 compared to the control. Collectively, the results show that excess L-Arg is sensed by the cell which then regulates the residual amount of amino acids concentration. The spectroscopy technique used here is highly sensitive, specific and accurate, can be readily automated and serves as a valuable tool for investigating the modulation of the arginine-nitric oxide pathway.

## Introduction

**Fig 1:**
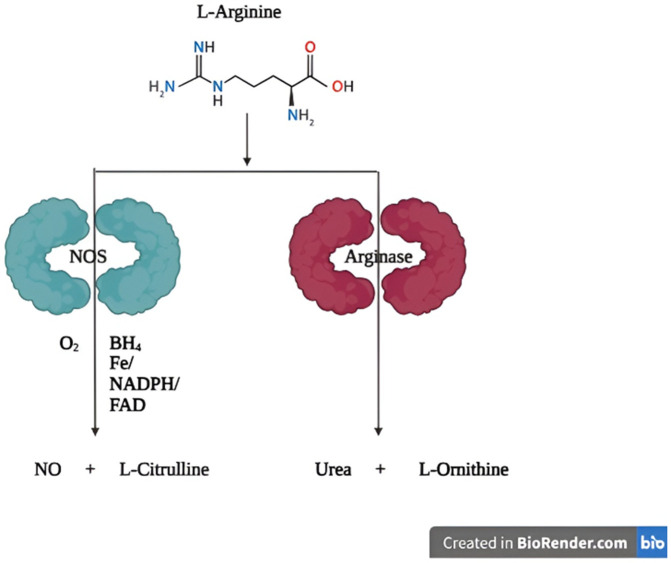
Schematic outlining L-arginine metabolism via nitric oxide synthase and arginase pathways. L-arginine is metabolized via two major enzymatic pathways. In the nitric oxide pathway, L-arginine is catalyzed by nitric oxide synthase (NOS), in the presence of essential cofactors, to produce nitric oxide (NO) and L-citrulline. Alternatively, L-arginine is hydrolyzed by arginase to yield L-ornithine and urea.

Amino acids play vital roles in animal biology, contributing to protein and glucose production, regulating cellular metabolism, supporting antioxidant defence mechanism, strengthening immunity and influencing gene expression either by activating or suppressing it [1]. L-Arginine is a semi-essential amino acid that plays a central role in diverse biological systems; ranging from protein synthesis to immune function and nitrogen metabolism [2]. As a metabolic hub, L-arginine serves as a precursor for critical pathways, including the generation of nitric oxide (NO), urea, polyamines, creatine, and other bioactive metabolites (Fig 1) [1,3]. There are primarily two enzymatic routes for catabolism of L-Arg. One is via Nitric oxide synthase (NOS), which catalyzes the oxidation of L-Arg to produce nitric oxide and L-Cit [4]. This reaction consumes NADPH and oxygen, generating NO; a versatile signaling molecule, involved in vascular tone regulation, neurotransmission, angiogenesis, immune response, and beyond [5,6]. The other route is via Arginase, which hydrolyzes L-Arg into L-Orn and urea, playing a pivotal role in the urea cycle and ammonia detoxification. L-Orn subsequently feeds into the biosynthesis of polyamines and other metabolites, while L-Cit can engage in recycling back into L-Arg, thereby contributing to nitrogen homeostasis [3,5].

Given the importance of L-arginine and its catabolic derivatives in both physiology and pathology; including immune regulation, cardiovascular function, and proliferative disorders; precise quantitation of these metabolites is essential for elucidating metabolic dynamics and pathway fluxes [5,7,8]. Analytically, high-performance liquid chromatography (HPLC) has been a long-standing methodological cornerstone for separating and quantifying amino acids like L-Arg, L-Orn, and L-Cit offering reliability and reproducibility [9,10]. More recently, advanced liquid chromatography-mass spectrometry (LC-MS) techniques have emerged, providing superior sensitivity, specificity, and structural resolution; especially valuable for detecting low-abundance metabolites and complex biological matrices [11,12].

Indeed, several studies have successfully developed methods enabling the simultaneous determination of L-Arg, L-Cit, and L-Orn using LC-MS/MS approaches, demonstrating enhanced throughput and analytical precision [13,14]. Primary amines in the amino acids are reacted with o-phthalaldehyde (OPA) and β-mercaptoethanol (ME) to form amino acid adducts that are then detected after separation by HPLC to determine amino acid concentrations [15,16]. Recent work has even enabled rapid quantification of underivatized amino acids; including arginine, ornithine, citrulline, and related methylated derivatives; in human culture supernatant via LC-MS platforms [17,18].

In this study, I propose a comprehensive analytical framework that leverages both HPLC and LC-MS to quantify the catabolic products of L-Arg; namely, L-Orn and L-Cit; as well as residual L-Arg. By employing this dual-platform strategy, I aim to evaluate the strengths and complementarities of these techniques, advancing our understanding of L-Arg metabolism and enabling accurate profiling of its metabolic fate across relevant biological contexts.

## Materials and methods

### Establishment of cell culture models for analysing catabolic products of L-arginine; L-ornithine and L-citrulline and the residual L-arginine

#### Culturing of adherent BNL CL2 and 3T3 L1 cell lines.

BNL CL2 and 3T3 L1 cell lines were used as model systems, obtained from two different sources. 3T3 L1 cells (RRID: CVCL_0123) were a generous gift from Dr Arcidiacono Biagio, University of Catanzaro ‘Magna Graecia’, Italy in 2019. BNL CL2 cells (RRID: CVCL_4383) were a generous gift from Associate Prof of Medicine Dimiter Avtanski, Zucker School of Medicine at Hofstra/Northwell, Hempstead, New York in 2019. The BNL CL2 cell line is a mouse hepatocyte epithelial insulin-sensitive cell [19,20], whilst the 3T3 L1 cell line is a mouse adipocyte (mouse embryonic fibroblast) insulin-sensitive cell [19,21]. The base medium for growth of both cell lines was Dulbecco’s Modified Eagle’s Medium (DMEM, Thermo Fisher Scientific, Cat. No: 41966). To prepare complete growth medium, foetal bovine serum (FBS, Sigma, Cat. No: F7524, non-USA origin) was added to a final concentration of 10% (v/v). During passaging, cells were maintained in vented T25 tissue culture flasks (Sarstedt, Germany) in complete growth medium (10 mL) and incubated in a static incubator (Thermo Forma, Thermo Fisher) at 37^o^C, 5% CO_2_. Both cell lines were routinely passaged every 3–4 days. For experiments, cells were used 2 or 3 days after passage when they were in growth phase.

### Preparation of stock solutions of L-arginine and growth medium with varying L-arginine concentrations

Commercially available DMEM media for Stable Isotope Labelling using Amino Acids (SILAC, Thermo Scientific, Cat. No: 88364), which is deficient in both L-lysine and L-arginine (**Table 1 in** S1 File), was used to generate growth medium with varying L-Arg concentrations. Foetal bovine serum (FBS, Sigma, Cat. No: F7524, non-USA origin) was added to a final concentration of 10% (v/v). L-lysine-HCl (Sigma, Cat. No: L-8662) was also added to give the same concentration of L-lysine (146 mg/L) as in the complete DMEM basal media (**Table 1 in** S1 File). This media was termed L-Arg deficient media, containing no exogenously added L-Arg (although FBS could contain unspecified concentrations of L-Arg (**Table 2 in** S1 File), the amount of L-Arg in the media was determined by HPLC after FBS addition as undetectable and hence this media was considered L-Arg deficient (0 µM) (**Table 3 in** S1 File). L-Arg (0.125 g, Sigma, A5006) was added to the SILAC L-Arg deficient DMEM media (10 mL) to obtain an L-Arg stock solution (71.75 mM). This was then diluted as required with the L-Lys and FBS containing SILAC media to generate different L-Arg concentration containing mediums.

### Establishment of cell culture models for analysing residual L-Arg and metabolites of L-Arg; L-Cit and L-Orn

BNL CL2 cells or 3T3 L1 cells were inoculated into a T125 tissue culture flask (Sarstedt, Germany) in complete DMEM (10% (v/v) FBS) in a total of 30 mL and the cells incubated for 2–3 days at 37^o^C, 5% CO_2_. Cells were then observed under a light microscopy (Zeiss, Germany) to confirm around 90–95% confluency, at which time the media was removed and cells were washed with pre-warmed (37^o^C) phosphate-buffered saline (PBS) (Oxoid, Cat. No: BR0014G). For trypsinisation of cells, pre-warmed 0.05% trypsin-EDTA (Gibco, Cat. No: 11580626, 3 mL) was added and incubated for 5 min at 37^o^C, 5% CO_2_ followed by addition of pre-warmed DMEM with 10% (v/v) FBS media (7 mL). A cell count was then performed using a Vi-CELL cell viability analyser (Beckman Coulter, Life sciences, USA) to determine viable cell concentrations (cells/mL) and culture viability (%). BNL CL2 or 3T3 L1 cells were then seeded into 6-well tissue culture plates (Greiner Bio-One) at 2x10^5^ viable cells/well in 2 mL of complete DMEM media.

**Fig 2:**
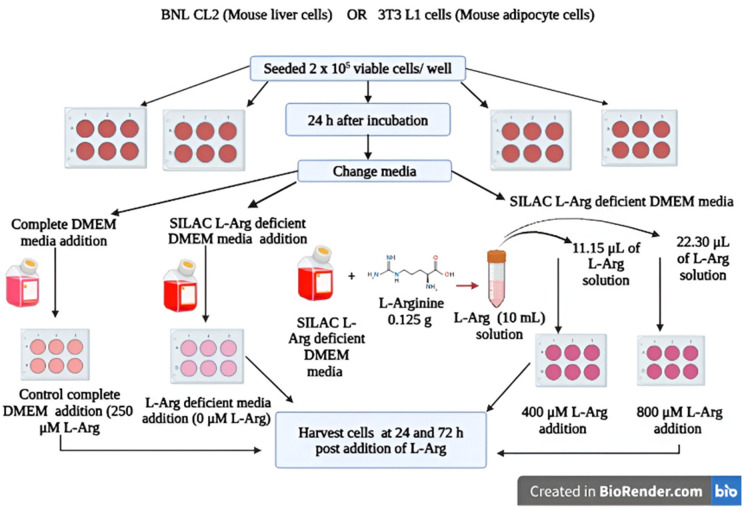
Schematic diagram outlining L-arginine supplementation of medium for BNL CL2 or 3T3 L1 cell growth. Cells were maintained in SILAC L-Arg deficient media supplemented with 0, 400 or 800 µM L-arginine or in control complete DMEM containing 250 µM L-arginine (2 mL/ well). Samples were collected at T = 0 (untreated, the cells were maintained in complete DMEM media for 24 h prior to the L-Arg addition experiment) and 24 and 72 h post addition of L-arginine for analysis.

The cells were then incubated in a static incubator (Thermo Forma, Thermo Fisher) for 24 h at 37^o^C, 5% CO_2_. After 24 h, the media was replaced with media of different concentrations of L-arginine (0, 400 and 800 µM) or the control complete DMEM with 10% (v/v) FBS (2 mL/ well) as in Fig 2. Complete DMEM is meant to contain 398.10 µM of L-arginine hydrochloride according to the media formulation from the company (Thermo Fisher) (**Table 1 in** S1 File), however this was measured as only 250 µM using the HPLC method used and described in this study (**Table 3 in** S1 File). Thus the 400 and 800 µM culture conditions represent a 1.6 and 3.2-fold increase in exogenous L-arginine concentration compared to the complete DMEM controls. Cell culture supernatant was then harvested at time points; 24 and 72 h post addition of L-Arg media of different concentrations. Cell culture supernatant was also harvested at T = 0; untreated samples (24 h after incubation of the cells and collected before L-Arg addition). Briefly, the cell culture media (2 mL) was collected from the cell cultures and frozen at -20^o^C to analyse metabolite of L-Arg in cell culture supernatant.

### Processing of biological samples

Samples were collected from L-arginine addition to BNL CL2 and 3T3 L1 cells as described above then vortexed and centrifuged at 1500 rpm for 10 min to obtain the cell free culture supernatant for the HPLC analysis.

### Deproteinization of samples for amnio acid analysis

Samples were deproteinised using a modified protocol of that described in previous research article [22]. To remove FBS and cellular proteins from the supernatant of the cell culture media, 100% (v/v) ice-cold ethanol (400 µL) was added to the cell culture supernatant (100 µL) followed by vertexing vigorously for 15 min at room temperature. Samples were then centrifuged at 17000 g for 15 min at 0°C. The supernatant (approximately 500 µL) was then transferred to a fresh microcentrifuge tube and the solvent and media removed by vacuum centrifugation in a SpeedVac-Plus SC110A concentrator centrifuge (Savant, USA) for 1.25 h at 45°C. The vacuum dried supernatant residue was then reconstituted in 80% (v/v) ethanol (100 µL) and vigorously vortexed for 5 min and then centrifuged again at 17000 g for 15 min at 0°C to collect the supernatant. To remove ethanol completely (to obtain high resolution of the amino acids and increase binding to the column in the HPLC experiment), the supernatant of resuspended sample (100 µL) was then further vacuum centrifuged by SpeedVac-Plus SC110A concentrator centrifuge (Savant, USA) for 30 min at 45^o^C and then 100 µL of water added.

### HPLC amino acid analysis

#### Pre-column derivatization.

**Fig 3:**
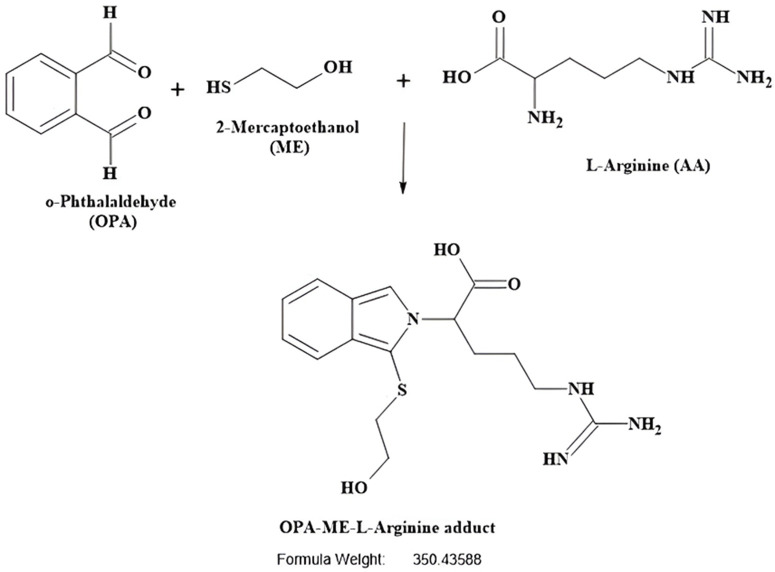
Chemical reaction between OPA and primary amino acid to form an adduct (OPA-ME-AA). Primary amino acid (AA) forms a derivative (OPA-ME-AA) with o-phthalaldehyde (OPA) in the presence of a thiol, 2-mercaptoethanol (ME). The adduct is well-resolved by gradient elution HPLC. The reaction takes place rapidly at room temperature at alkaline pH.

Amino acids were derivatized at room temperature using a pre-column derivatisation method. O-phthaldialdehyde (OPA) reagent complete solution (Sigma, Cat. No: P0532) consisting of OPA (1 mg/mL), Brij^R^ 35 (for optimum separation of citrulline from threonine and of ornithine from lysine [15]), methanol, 2-mercaptoethanol, potassium hydroxide and boric acid, pH 10.4, was used as a pre-column derivatization agent, specially formulated for primary amines and amino acids at alkaline pH. OPA reacts with primary amino acids in the presence of a thiol (2-mercaptoethanol) to form adducts (OPA-ME-AA) (Fig 3), which are well-resolved by HPLC with appropriate gradient conditions and mobile phases with organic modifiers [23].

### Mobile phase buffers

Mobile phase A (0.1 M sodium acetate, pH 7.2) was prepared as described in previous study [15]. In brief, sodium acetate (0.1 M) (Sigma ultra, 99.0%, Cat. No: S7545), 9% methanol, and 0.5% tetrahydrofuran (ACROS Organics, 99.5%, Cat. No:10292182), adjusted to pH 7.2 by HCl, was prepared and mixed after each addition before being filtered through a 0.2 μm nylon membrane filter (MERCK, Cat. No GNWP04700) prior to use in HPLC analysis. The mobile phase B was 100% (v/v) methanol.

### Preparation of amino acid standards

A standard amino acid mixture (6 mM), consisting of the essential 20 amino acids, was a gift from Dr Andrew Lawrence, School of Biosciences, University of Kent, and was prepared as outlined previously [24].^.^ In brief, the standard amino acid powder mixture (21 L-amino acids and glycine, Sigma, Cat. No: 09416−1EA) was dissolved at an alkaline pH to formulate a stock solution of standard amino acid mixture (6 mM) and then diluted to obtain a working amino acid standard (0.6 mM for each amino acid). Additionally, L-citrulline (Sigma, Cat. No: C7629) and L-ornithine monohydrochloride (Sigma, Cat. No: O2375) (0.6 mM of each) were added to the standard amino acid mixture from respective stock solution of L-citrulline and L-ornithine (6 mM of each) to form an extended amino acid standard solution.

### Amino acid HPLC analysis

An Agilent 1100 HPLC (Agilent Technologies, Germany) equipped with a diode-array detector (DAD) was used. HPLC experimental conditions were optimised (OPA derivatisation method, column, detection parameters and DAD detector) to obtain well-resolved peaks for the intended amino acids. Samples were injected (25 μL) onto an ACE HPLC column; RP-C18, dimensions 125 × 4.6 mm, 5 μm (Cat. No: ACE 126–1246). The column was operated with a flow rate of 1.1 mL/min using organic modifiers; 0.1 M sodium acetate, pH 7.2 as mobile phase A and 100% methanol as mobile phase B. Before running the sample set, the column was fluxed sequentially with water and methanol for 5 min and then equilibrated with mobile phase A for 10 min at a flow rate of 1.1 mL/min at 30°C.

To a dark glass vial (1.5 mL), an extended amino acid standard mixture (0.6 mM each) or test cell culture sample solution (100 µL) was added. The autosampler was automated to mix 25 µL of a standard or sample solution with 25 µL of the OPA reagent solution and allowed for incubation for 1 min in a reaction loop. The derivatized solution was then immediately delivered into the HPLC column without any delay as the amino acid derivatives are very unstable, with short half-lives (2.3–72.3 min) [23]. This problem was overcome by automating the derivatization and replacing the OPA micro vial often. Amino acids were separated with linear gradient as described in the Table 1 with a total running time of 49 min; flow rate, 1.1 mL/min. The detector, DAD, was programmed to switch to 338 nm, 10 nm bandwidth, and reference wavelength 390 nm, 20 nm bandwidth. The molar absorptivity of each derivatized amino acid was detected at 338 nm (λmax).

**Table 1:** HPLC gradient program for separation of L-arginine, L-citrulline and L-ornithine at flow rate 1.1 mL/min.

Mobile Phase (%)	Time (min)										
	0	15	20	24	26	34	38	40	42	42.1	49
**A**	86	86	70	65	53	50	30	0	0	86	86
**B**	14	14	30	35	47	50	70	100	100	14	14

### Standard curve generation for L-arginine, L-citrulline and L-ornithine

Stock solutions of L-arginine, L-citrulline and L-ornithine (20 mM, 10 mL each and 1000 µM, 50 mL each) were diluted to give a range of concentrations of each amino acid standard (10, 20, 30, 40, 50, 100, 250, 500, 750 and 1000 µM). The range of standard solutions were run with OPA on the HPLC. Peaks in the HPLC chromatogram were integrated using the software provided by the manufacture (Agilent Technologies, Germany) to obtain the area of the peaks and from these a standard curve was plotted with the area of the signals (mAU x Sec) against concentration to obtain best fit standard curves for each amino acid.

### HPLC data analysis

Identification of particular amino acid signals was based on the comparison between the retention time of the extended amino acid standard mixture and the amino acids of interest from analysis of samples. Quantitation was based on the standard curve method using a linear curve fitted by linear regression analysis. The unknown concentration of each amino acid in experimental samples was calculated using the equation of the line fitted for the standard curves of amino acids.

### Analysis of L-arginine, L-citrulline and L-ornithine adducts with OPA derivatising agent by liquid chromatography – mass spectrometry (LC-MS)

LC-MS (UltiMate 3000 HPLC and UHPLC Systems, Thermo Fisher) was carried out to confirm the molecular mass of the L-arginine, L-citrulline and L-ornithine adducts after derivatisation with the derivatisation agent o-phthalaldehyde (OPA) containing β-mercaptoethanol. Mobile phase modifiers were 0.1% (v/v) formic acid and H_2_O (mobile phase A) and 0.1% (v/v) formic acid and methanol (mobile phase B). An autosampler was programmed to inject a sample volume 20 µL into the analytical column (C18; 4.6 mm x 150 mm, 5 µm, from Zorbax Ecliplse), guarded with SecurityGuard™ ULTRA Cartridges UHPLC column (C18; 2 mm, 2.1 mm). Before running a sample, the column was washed sequentially with 2% mobile phase B and then equilibrated with 2% mobile phase B for 13 min before the first sample at a flow rate of 1.5 mL/min and constant column temperature of 40^o^C. The amino acid adducts were resolved with the gradient described in Table 2. The signals were detected at a wavelength of 220 nm. Mass spectrometry was undertaken in positive mode [MH]^+^ (L-arginine, L-citrulline and L-ornithine) and negative mode [M-H]^-^ (L-arginine) to confirm the presence of the expected adducts.

**Table 2:** LC-MS gradients used to identify the derivatisation adducts of amino acids (OPA-ME-AA) at a flow rate 1.5 mL/min.

Time (min)	1	> 8	2.5	2.5
**Gradient Mobile Phase B %**	2	2-100	100	2

### Statistical methods

Statistical analysis was undertaken using GraphPad Prism 9.4.1 software and Microsoft Excel. Samples were analysed in triplicate biological replicates. Data interpreted of means and standard deviation were analysed using two-way ANOVA. The Tukey’s multiple comparison method was used to determine differences among the means of the treatment groups (0, 400 and 800 µM L-Arg) with the control complete DMEM media addition and untreated samples at T = 0 across the time points 24 and 72 h. Probability values ≤ 0.05 were considered to indicate statistical significance. The stars approach intended to flag levels of significance were followed (American Psychological Association style, New England Journal of Medicine) as ns = P > 0.05, * = P ≤ 0.05, ** = P ≤ 0.01, *** = P ≤ 0.001 and **** = P ≤ 0.0001.

## Results

### Confirmation of the formation of the expected L-arginine, L-ornithine and L-citrulline adducts for amino acid analysis by liquid chromatography – mass spectrometry (LC-MS)

Before undertaking the amino acid analysis by HPLC, mass spectrometry was undertaken to ensure that the expected adducts were formed and detected for analysis. Primary amines in the amino acids are reacted with o-phthalaldehyde (OPA) and β-mercaptoethanol (ME) to form amino acid adducts that are then detected after separation by HPLC to determine amino acid concentrations. The expected mass and the experimental mass measured of the OPA-ME-amino acid adducts are described in Table 3.

**Table 3:** Comparison of expected mass of the adducts with the experimental mass.

Adducts	Expected mass of the adducts	Experimental mass measured in positive polarity [MH]^+^	Experimental mass measured in negative polarity [M-H]^-^
OPA-ME-L-arginine	350.16	351.44	349.00
OPA-ME-L-citrulline	351.19	351.40	Not determined
OPA-ME-L-ornithine	308.12	309.40	Not determined

**Fig 4:**
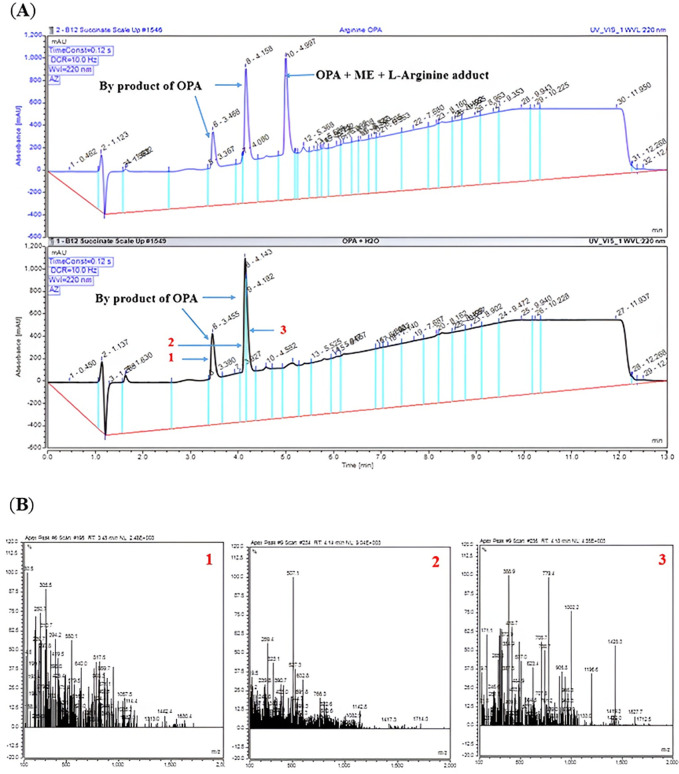
LC chromatogram with the signals observed for L-arginine with OPA and mass spectrum for OPA in water. Three signals appeared for L-arginine and OPA reagent in the LC trace (**A**, top panel**)**. The two signals on the left are combination products of OPA and a signal on the right is for the OPA-ME-L-arginine adduct. Signals for OPA in water alone on the LC appeared as two peaks (**A**, bottom panel**)**. Mass spectra data for OPA in water (**B**: 1, 2 and 3**)** shows multiple combination products are generated from OPA.

**Fig 5:**
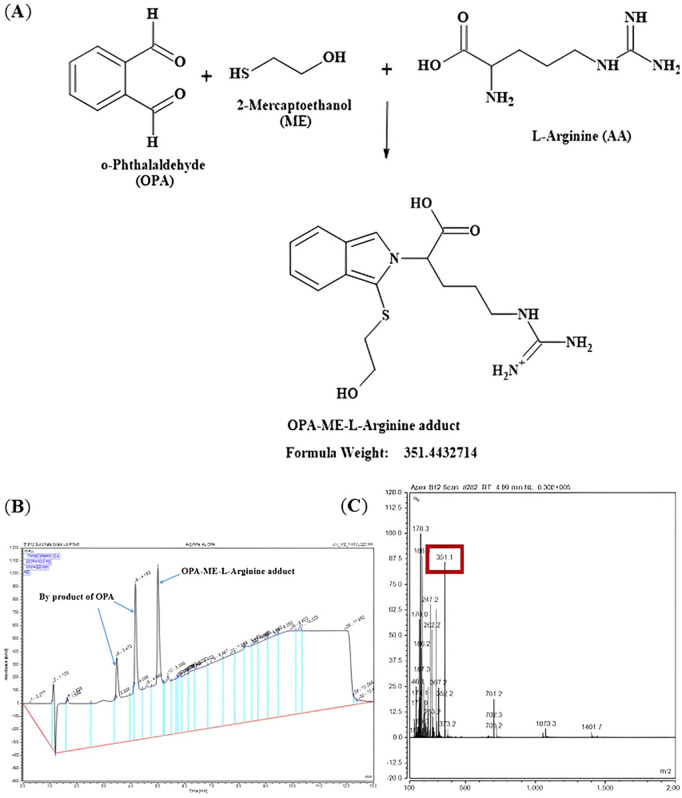
Schematic reaction mechanism and mass spectrum for formation of L-arginine adduct in positive polarity. **(A)** Reaction schematic for formation of L-arginine adduct (isoindole derivative) and expected mass with positive polarity [MH]^+^ by reacting with the derivatizing agent OPA containing ME. **(B)** LC chromatogram with the signals observed for L-arginine with OPA absorbance at 220 nm. The two peaks on the left are derived from OPA or other products of OPA and the single peak on the right is for the expected OPA-ME-L-arginine adduct. **(C)** Mass spectra with a protonated molecule detected at a base peak at m/z 351.1 for the diagnostic fragment ion for the OPA-ME-L-arginine adduct.

**Fig 6:**
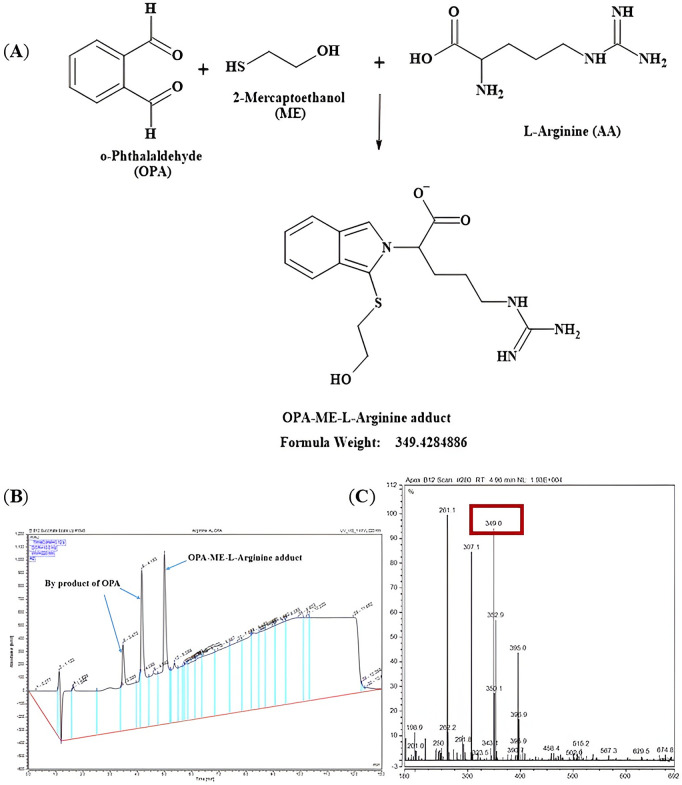
Schematic reaction mechanism and mass spectrum for formation of L-arginine adduct in negative polarity. **(A)** Reaction schematic for formation of L-arginine adduct (isoindole derivative) and expected mass with negative polarity [M-H]^-^ by reacting with the derivatizing agent OPA containing ME. **(B)** LC chromatogram with the signals observed for L-arginine with OPA absorbance at 220 nm. The two peaks on the left are derived from OPA or other products of OPA and the single peak on the right is for the expected OPA-ME-L-arginine adduct. **(C)** Mass spectra with an ionized molecule detected at a base peak at m/z 349.0 for the diagnostic fragment ion for the OPA-ME-L-arginine adduct.

The formation of the correct L-arginine adduct was confirmed by LC-MS. In the liquid chromatography chromatogram (Fig 4 (A), top panel) there were three signals observed, the two peaks on the left side (retention times 3.5 and 4.25 min) are due to the OPA and by products and combination products of OPA, whilst the single peak on the right (retention time 5 min) was confirmed as the formation of the expected L-arginine adduct (OPA-ME-L-Arg) by mass spectrometry (experimental mass m/z 351.44 in positive polarity, Fig 5 (C) and experimental mass m/z 349.0 in negative polarity, Fig 6 (C). The two peaks derived from OPA were also confirmed by the LC-MS data of OPA and water (Fig 4 (A), bottom panel). The mass spectral information providing additional confirmation of the multiple fragments from the OPA in water is presented in Fig 4 (B).

**Fig 7:**
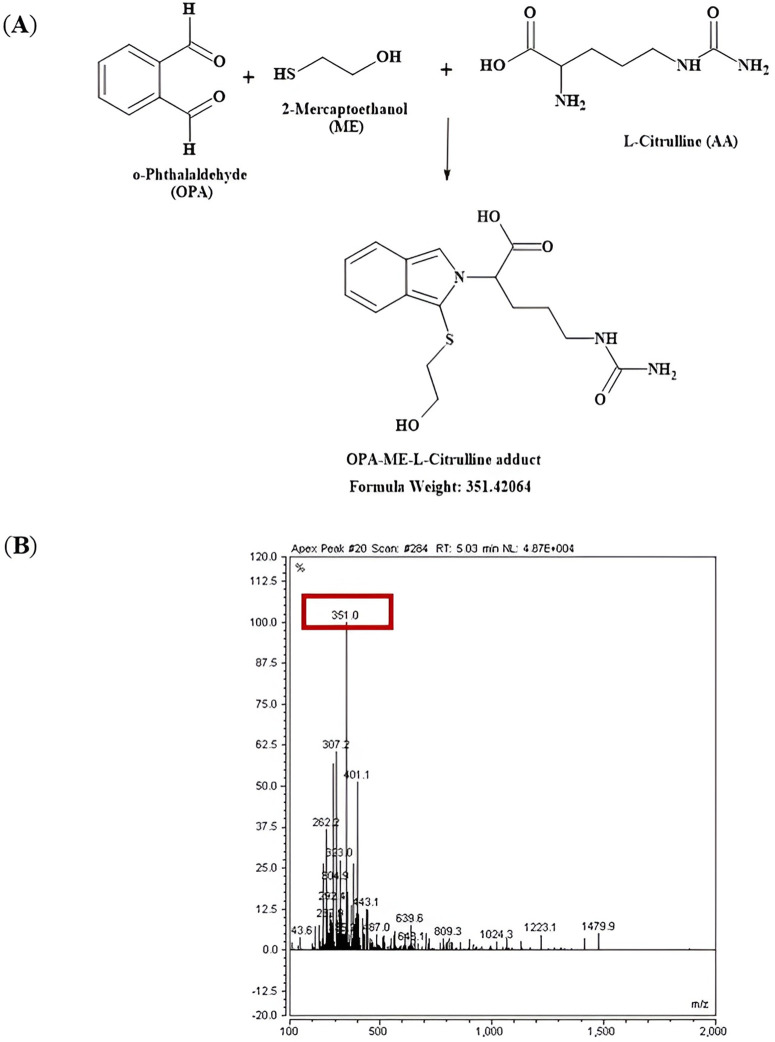
Schematic reaction mechanism, HPLC chromatogram and mass spectrum for formation of L-citrulline adduct in positive polarity. **(A)** Reaction schematic for formation of L-citrulline adduct (isoindole derivative) and expected mass with positive polarity [MH]^+^ by reacting with the derivatizing agent OPA containing ME. **(B)** Mass spectra showing a protonated molecule detected at a base peak at m/z 351.0 for the diagnostic fragment ion for the OPA-ME-L-citrulline adduct.

**Fig 8:**
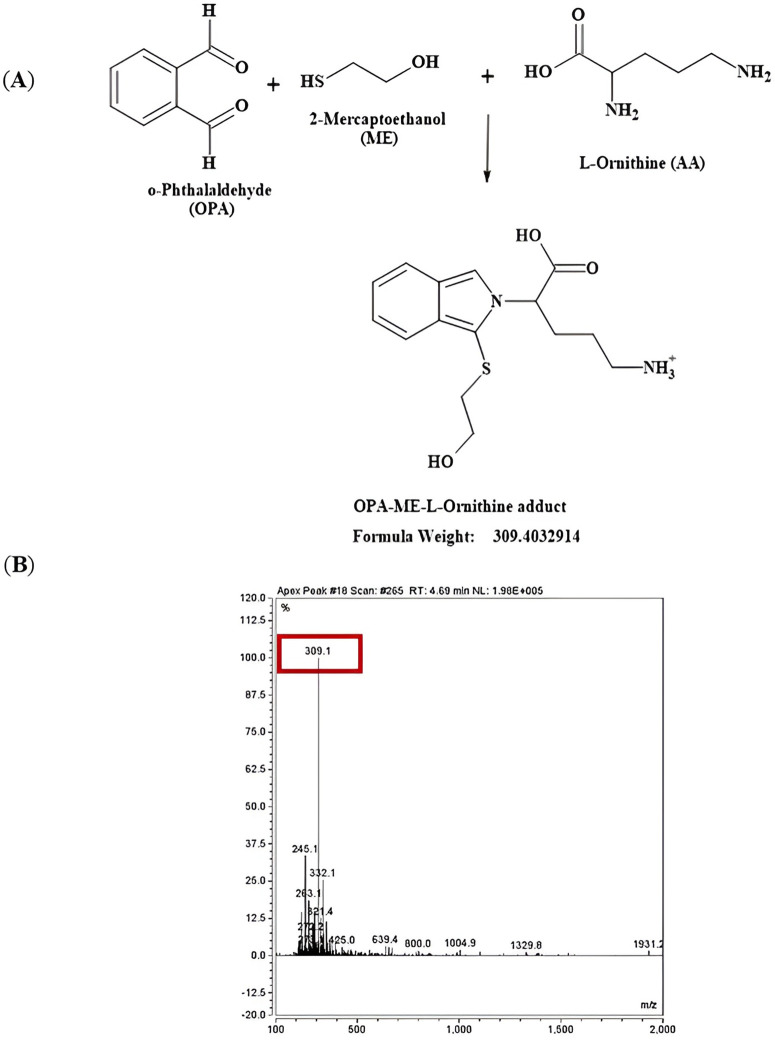
Schematic reaction mechanism, HPLC chromatogram and mass spectrum for formation of L-ornithine adduct in positive polarity. **(A)** Reaction schematic for formation of L-ornithine adduct (isoindole derivative) and expected mass with positive polarity [MH]^+^ by reacting with the derivatizing agent OPA containing ME. **(B)** Mass spectra showing a protonated molecule detected at a base peak at m/z 309.1 for the diagnostic fragment ion for the OPA-ME-L-ornithine adduct.

Switching of polarity between positive polarity [MH]^+^ (Fig 5**, Fig A in** S1 File) and negative polarity [M-H]^-^ (Fig 6) further confirmed that the L-arginine adduct (OPA-ME-L-Arg) was formed during the pre-column-derivatization process. The formation of OPA-ME-amino acid adduct was confirmed for the other amino acids of interest too, L-citrulline (Fig 7**, Fig A in SI File**) and L-ornithine (Fig 8**, Fig A in** S1 File) by LC-MS.

### HPLC analysis of residue L-arginine, L-ornithine and L-citrulline in the cell culture media of BNL CL2 cells cultured in different initial L-arginine concentrations

**Fig 9:**
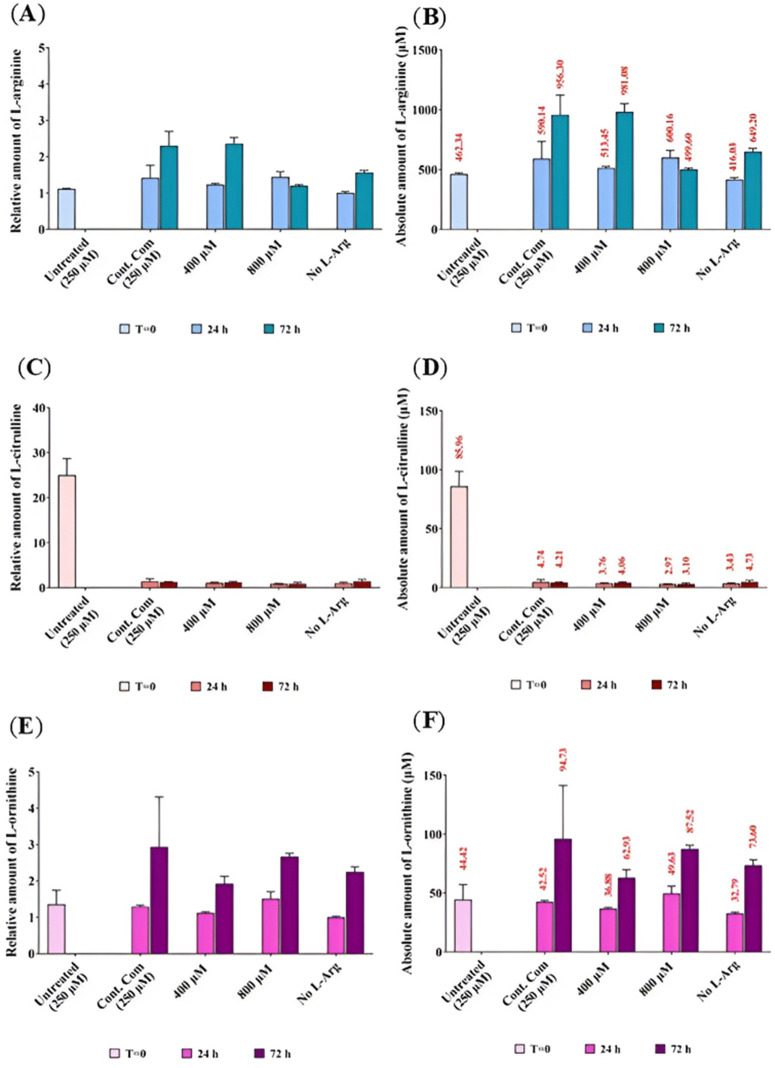
Relative and absolute quantification of residual serum L-Arg, L-Cit and L-Orn in BNL CL2 cells cultured in different L-Arg concentrations and the control for 24 and 72 h. Relative **(A)**, **(C)** and **(E)**, respectively) and absolute **(B)**, **(D)** and **(F)**, respectively) residue amount of L-Arg, L-Cit and L-Orn analysed in culture supernatant of BNL CL2 cells cultured in different amount of L-Arg (0, 400 and 800 µM) in L-Arg deficient media and the control complete DMEM media (containing 250 µM L-Arg) for 24 and 72 **h.** Untreated samples were maintained in complete DMEM media (containing 250 µM L-Arg) and collected 24 h after the incubation prior to L-Arg addition experiment (T = 0). Data points represent the mean ± SD of each sample. Error bars represent the standard deviation from the mean (n = 3). Tables summarise two-way ANOVA followed by a Tukey multiple comparison test using GraphPad Prism 9.4.1. The stars indicate the levels of significance; ns = P > 0.05, * = P ≤ 0.05, ** = P ≤ 0.01, *** = P ≤ 0.001 and **** = P ≤ 0.0001.

As described in the introduction chapter, L-arginine is oxidised and produces L-citrulline and NO, this pathway is catalysed by NOS. Calculated residual concentration of selected amino acids, L-Arg, L-Cit and L-Orn in experimental samples was undertaken using the equation of the line fitted for standard amino acid samples to give the absolute concentration of L-Arg, L-Cit and L-Orn present in the BNL CL2 (Fig 9(A), (**C**) and (**E**), respectively). The absolute concentration of each amino acid was then normalized to the absolute concentration of the respective amino acid present in no L-Arg cultures (0 µM) at 24 h to obtain the relative concentration of selected amino acids L-Arg, L-Cit and L-Orn in BNL CL2 (Fig 9(B), (**D**) and (**F**), respectively).

When investigating L-Arg present in the supernatant of cultured cells (Fig 9 (**A**) and (**B**) the amount of L-Arg increased (P < 0.0001) in the samples cultured in control complete DMEM (956.3 µM) and 400 µM L-Arg (981.1 µM) at 72 h time point. Cell samples cultured in no L-Arg at 72 h also showed high (P < 0.0001) amounts of L-Arg (649.19 µM). Arginine at 400 µM had decreased (523 µM) amount of L-Arg at 24 h compared to the control (590 µM). The opposite profile was observed in samples cultured in 800 µM L-Arg, which was increased (600 µM) in the amount of L-Arg at 24 h compared to the control complete DMEM (590 µM) after which this was decreased (499 µM) at 72 h compared to the control (956 µM).

Metabolism of arginine occurs in the liver to produce urea [25]. Arginase activity is high in liver to facilitate the urea cycle, therefore to maintain the nutritional status of arginine (bioavailability of arginine in the human body) by dietary intake, there is an arginine-citrulline-arginine cycle within the major organs or systems; intestine, liver and kidneys. The amino acid content in liver cells is thus mainly regulated by the urea cycle [26–30].

Arginine is synthesized from citrulline in almost all cell types [31]. Amino acid homeostasis including amino acid uptake, *de-novo* synthesis and recycling impacts the availability of each amino acid in the culture medium. It was expected that over time the fate of amino acids such as depletion at different rates, oxidation, release from dying cells and synthesise and release/secretion of some amino acids will impact the concentration of amino acids in the culture medium [32]. L-glutamine and L-arginine are both conditionally essential amino acids. In *de-novo* synthesis of amino acids, for example, glutamine is an amino acid that can be used as a precursor to synthesis L-citrulline [33]. The culture media used in this study contained high glutamine concentrations (**Table 1 in** S1 File; L-glutamine in complete DMEM; 3972.6 µM and no L-arginine SILAC DMEM; 3995.9 µM) compared to other amino acids presented. Therefore, we analysed the residual amounts of selected amino acids presented in the culture medium and compared it to the initial amounts or compared it across the time period.

In most of the cell supernatant samples the amount of arginine had increased. When L-Arg was analysed by the HPLC, the L-Arg amount was high in 400 µM at 72 h (981.1 µM) and 800 µM at 24 h (600.16 µM) compared to the control complete DMEM cultures at 24 (590.14 µM) and 72 h (956.3 µM). This may be the residual amount of L-Arg or this had come from cells either secreting arginine or dead cells and turnover of protein as the system is closed.

When analysing L-Cit present in the different cultured conditions (Fig 9 (C) and (**D**), surprisingly, untreated sample at T = 0 showed the highest amount of L-Cit (85.96 µM) (P < 0.0001) among all samples. This may reflect that the culture growth media contained very much high concentrations of glutamine, which could be used as a precursor to synthesis L-Cit at T = 0 (**Table 1 in** S1 File) although how this would occur at T = 0 is unknown. The second highest amount of L-Cit was in samples cultured in control complete DMEM at 24 h (4.74 µM) and no L-Arg added at 72 h (4.73 µM). It was noted that the amount of L-Cit present in the L-Arg treated samples was independent of culture time, but dependent on the amount of exogenously added L-Arg, because the levels of L-Cit in L-Arg at 400 and 800 µM after 24 (400 µM; 3.76 µM and 800 µM; 2.97 µM) and 72 h (400 µM; 4.1 µM and 800 µM; 3.1 µM) were more or less the same (P < 0.0001). The control had a high amount of L-Cit (P < 0.0001) compared to the L-Arg treated samples cultured in 400 and 800 µM at 24 (0.79-fold and 0.62-fold, respectively) and 72 h (0.97-fold and 0.74-fold, respectively).

When investigating the amount of L-Orn present in the cultured samples (Fig 9 (**E**) and (**F**), there was an overall increase (P < 0.0001) in the amount of L-Orn across the time points in all samples. However, the control samples cultured in complete DMEM had the highest amount of L-Orn (42.52 µM) (P < 0.0001) compared to all the samples except L-Arg at 800 µM (24 h; 49.63 µM) and untreated at T = 0 (44.42 µM). Samples cultured in L-Arg at 400 µM contained decreased (P < 0.0001) amounts of L-Orn at 24 (0.87-fold) and 72 h (0.655-fold). However, in samples cultured in L-Arg at 800 µM there was increased (P < 0.0001) amounts of L-Orn present at 24 (1.17-fold) and 72 h (0.91-fold). Very similar fold change (0.77-fold) was observed in the samples cultured in 0 µM L-Arg at 24 and 72 h compared to the control at the same time points.

HPLC analysis of residue L-arginine, and L-ornithine and L-citrulline in the cell culture media of 3T3 L1 cells cultured in different initial L-arginine concentrations

**Fig 10:**
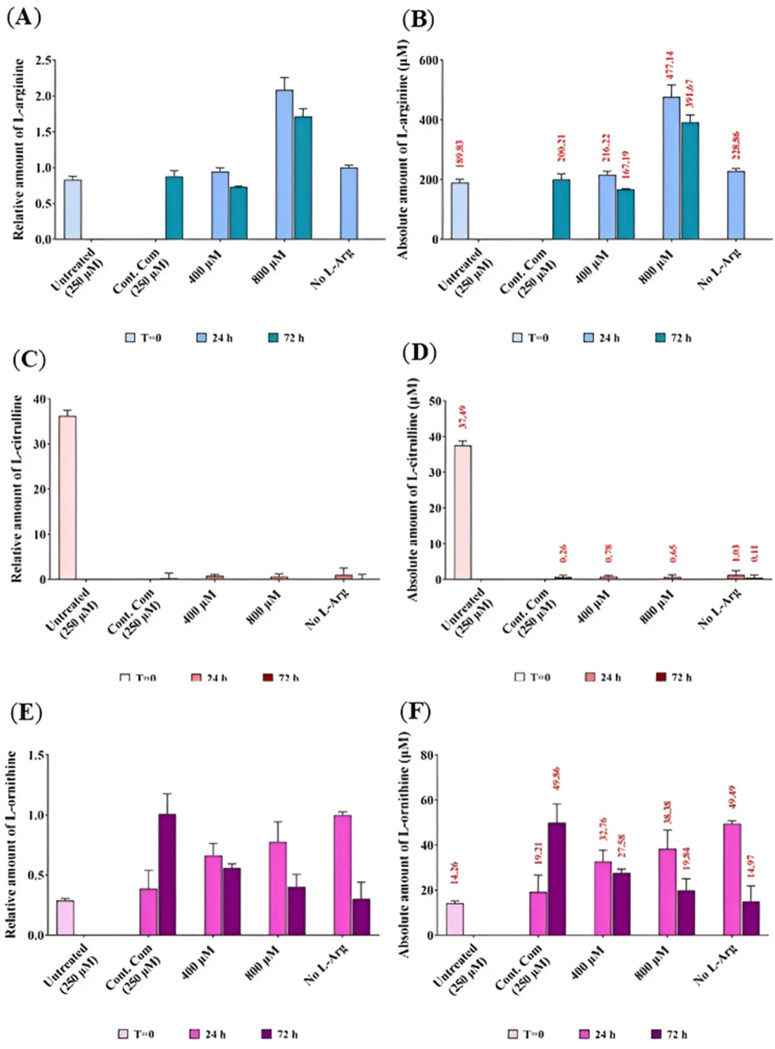
Relative and absolute quantification of residual L-Arg, L-Cit and L-Orn in culture medium of 3T3 L1 cells cultured in different L-Arg concentrations and the control for 24 and 72 h. Relative **(A),** **(C)** and **(E)**, respectively) and absolute **(B),** **(D)** and **(F)**, respectively) residue amount of L-Arg, L-Cit and L-Orn analysed in culture supernatant of 3T3 L1 cells cultured in different amount of L-Arg (0, 400 and 800 µM) in L-Arg deficient media and the control complete DMEM media (containing 250 µM L-Arg) for 24 and 72 h**.** Untreated samples were maintained in complete DMEM media and collected 24 h after the incubation prior to L-Arg addition experiment (T = 0). Data points represent the mean ± SD of each sample. Error bars represent the standard deviation from the mean (n = 3). Tables summarise two-way ANOVA followed by a Tukey multiple comparison test using GraphPad Prism 9.4.1. The stars indicate the levels of significance; ns = P > 0.05, * = P ≤ 0.05, ** = P ≤ 0.01, *** = P ≤ 0.001 and **** = P ≤ 0.0001.

The absolute (Fig 10 (**A**), (**C**) and (**E**), respectively) and relative (Fig 10 (**B**), (**D**) and (**F**), respectively) concentrations of L-Arg, L-Cit and L-Orn were investigated in the samples. The amount of L-Arg in cultured 3T3 L1 samples was investigated by HPLC (Fig 10 (**A**) and (**B**). L-Arg was not detectable in samples cultured in the control complete DMEM at 24 h. The amount of L-Arg decreased (P < 0.0001) with culture time in 400 and 800 µM L-Arg at 24 (216.22 µM and 477.14 µM, respectively) and 72 h (167.19 µM and 391.67 µM, respectively). At 72 h, L-Arg decreased at 400 µM (167.19 µM) but increased at 800 µM (391.67 µM) compared with the control at 72 h (200.21 µM).

In the investigation of L-Cit (Fig 10 (**C**) and (**D**), untreated samples at T = 0 had the highest (P < 0.0001) amount of L-Cit (37.49 µM) among all samples. There was no detectable amount of L-Cit present in some cultured samples. The amount of L-Cit present in 800 µM L-Arg was higher (1.13-fold) (P < 0.0001) compared to the control. At 24 h, there was no detectable amount of L-Cit present in the cultured samples except for samples cultured in no L-Arg (0.26 µM).

For the analysis of L-Orn (Fig 10 (**E**) and (**F**), samples cultured in control complete DMEM had the highest (P < 0.0001) amount of L-Orn (49.86 µM) among all samples. The amount of L-Orn was decreased (P < 0.0001) in samples cultured in L-Arg at 400 (at 24 h; 32.76 µM and 72 h; 27.58 µM) and 800 µM (at 24 h; 38.38 µM and 72 h; 19.84 µM) across the time points. However, L-Orn was increased in the control complete DMEM (at 24 h; 19.21 µM and 72 h; 49.86 µM) across the time points.

## Discussion

Large amounts of branched chain amino acids (BCAAs) are metabolised in adipose tissue [34]. An isotopic labelled study reported that in adipose tissue insulin and the amount of glucose in the medium play a role in the integration of glycine, L-proline and histidine [35]. The role of white adipose tissue (WAT) in amino acid metabolism has been described and the studies predict that WAT consists of a completely functional urea cycle [36].

In this study, in 3T3 L1 cultured cell supernatants L-Arg was high in L-Arg treated cell samples after 24 h compared to the control, but decreased after 72 h. A previous study demonstrated a remarkable pathway of L-arginine, L-ornithine and L-citrulline, which are involved in urea cycle of white adipose tissue [36]. From the urea cycle, it is proposed from the data that exogenously added excess L-Arg might not be used for the synthesis of L-Orn but rather be utilised by enzymes involved in L-Arg metabolism. L-Arg is a substrate for nitric oxide synthase and arginase enzyme which produces L-Cit and L-Orn, respectively [37]. It has been reported that Km values for arginine of arginase I and II (around 10 mmol/L) are significantly higher than that of iNOS (around 5 µmol/L) [38]. This reflects that arginase needs a lot of L-Arg substrate to actively involve in the synthesis of L-Orn. It is suggested that when excess L-Arg (400 and 800 µM) was added to 3T3 L1 cells, the substrate L-Arg concentration was higher than the enzymatic activity of arginase therefore 3T3 L1 cells cultured in excess L-Arg did not show high cell culture supernatant L-Orn concentrations. This also assumes that L-Orn could be secreted and thus investigating intracellular amounts would be interesting in the future.

The arginine-derived products by the NOS and arginase enzyme-catalysed reactions were investigated by HPLC to quantify residual amino acids. HPLC can be employed to identify directly (detect the derivatised amino acid adduct) the products of arginine metabolism. In some cells or tissues, especially in liver cells, however, other pathways of arginine metabolism are present and the products formed may fail to be separated from arginine, from each other, or from citrulline or ornithine using the HPLC method described in this work. For example, the widely distributed enzyme arginase can hydrolyze arginine to ornithine and urea. Using the HPLC method, it is not possible to distinguish ornithine from arginine or citrulline from arginine. Monitoring conversion of radiolabelled arginine to citrulline, the co-product of the nitric oxide synthase (NOS)-catalysed reaction or arginine to ornithine, the co-product of arginase catalysed reaction would distinguish the catabolic products of L-Arg. Therefore, for residual amino acids analysis radiolabelled L-Arg can be used to identify the amount of L-Cit or L-Orn produced from L-Arg metabolic pathway using ion-exchange separation. This separation procedure allows quantification of the relative activities of NOS and arginase in cell and tissue extracts by permitting identification of radiolabelled arginine-derived citrulline and/or ornithine. This method is useful, therefore, as a means to identify arginine-derived citrulline and/or ornithine, to assess the contribution of pathways other than that catalysed by NOS to the generation of arginine-derived products, and to simultaneously monitor NOS and arginase activities in the cells. Further, ornithine and proline are the major products of arginine catabolism via the arginase pathway [39]. Analysing the residue proline could also help explain the effect of arginine on metabolism in these cell types. Whether ornithine and/or proline affect metabolic pathway through arginase enzyme in BNL CL 2 hepatocytes and 3T3 L1 adipocytes remains to be determined.

There has been interest in assessing whether delivery of specific amino acids can improve patient outcome under various pathological conditions [40]. L-glutamine is the most plentiful amino acid in the human body and plays an important role in catabolic states and is a crucial factor in various cellular and organ functions [40]. L-glutamine and L-arginine, conditionally essential amino acids, are able to work together to improve inflammation response [41]; however, L-arginine reduced L-glutamine’s antioxidant properties [42]. Although there is a lack of research on the combined effects of L-arginine and L-glutamine, the preliminary data shows that combining the two may benefit those with intestinal inflammation in cultured colonic biopsies from patients with active Crohn’s disease (CD) [41]. In the current study, one of the products of L-Arg/NOS pathway; L-Cit was unexpectedly very much high in untreated samples at T = 0, when the cells were grown in complete DMEM media, which formulated with high concentration of Glutamine (**Table 1 in** S1 File). The present study was undertaken to evaluate the effects of an L-Arg, in future comparing this with supplementation with L-glutamine and L-alanine (the latter as a negative control) might be useful to analyse if there is a combination effect of these amino acids in BNL CL2 and 3T3 L1 cells.

L-Arg may be useful as an exercise supplement in different capacities. It has been shown that L-Arg can prolong exercise ability in people suffering from congestive heart failure [43]. In order to increase blood flow to working muscles, L-Arg acts as a vasodilator [44]. So, these properties may also increase exercise duration in healthy individuals. The L-Arg supplement may be effective at increasing growth hormone levels [45], so it may not only be beneficial for exercise but also to promote good health in general. There is no official recommended daily allowance (RDA) for L-Arg. Our best understanding is that 2–3 grams of L-Arg per day is completely safe dose for a healthy individual to achieve the benefits of L-Arg and maintain good health. Who knows, a novel, safe and effective means of preventing and treating obesity in mammals may potentially be supported by controlling arginine supplementation.

## Supporting information

S1 File Supplemental Tables, Figures, and Captions. (PDF)

S2 File Statistical Tables. (PDF)

## References

[1] Sah N, Wu G, Bazer WB. Amino acids in nutrition and health. Amino acids in gene expression, metabolic regulation, and exercising performance. Springer. 2021. p. 7–21. doi: 10.1007/978-3-030-74180-8

[2] Fung TS, Ryu KW, Thompson CB. Arginine: at the crossroads of nitrogen metabolism. EMBO J. 2025;44(5):1275–93. doi: 10.1038/s44318-025-00379-3 39920310 PMC11876448

[3] Wu G, Morris S. Amino Acids in Nutrition and Health. Biochem J. 2008;17:1–17. doi: 10.1007/978-3-030-45328-2

[4] Morris SM Jr. Enzymes of arginine metabolism. J Nutr. 2004;134(10 Suppl):2743S-2747S; discussion 2765S-2767S. doi: 10.1093/jn/134.10.2743S 15465778

[5] Morris SM Jr. Arginine Metabolism Revisited. J Nutr. 2016;146(12):2579S-2586S. doi: 10.3945/jn.115.226621 27934648

[6] Morris SM Jr. Arginine metabolism: boundaries of our knowledge. J Nutr. 2007;137(6 Suppl 2):1602S-9S. doi: 10.1093/jn/137.6.1602S 17513435

[7] Kurhaluk N, Tkaczenko H. L-Arginine and Nitric Oxide in Vascular Regulation-Experimental Findings in the Context of Blood Donation. Nutrients. 2025;17(4):665. doi: 10.3390/nu17040665 40004994 PMC11858268

[8] Li S, Ye X, Wen X, Yang X, Wang L, Gao K, et al. Arginine and its metabolites stimulate proliferation, differentiation, and physiological function of porcine trophoblast cells through β-catenin and mTOR pathways. BMC Vet Res. 2024;20(1):167. doi: 10.1186/s12917-024-04023-w 38689278 PMC11062007

[9] Ridwan R, Razak HRA, Adenan MI, Saad WMM. Separation of L-arginine and L-citrulline in red and yellow crimson watermelon (Citrullus lanatus) juices extract using HPLC gradient mode. Malaysian J Anal Sci. 2018;22(5):785–93. doi: 10.17576/mjas-2018-2205-06

[10] Ahmed R. High-performance liquid chromatography (HPLC): principles, applications, versatility, efficiency, innovation and comparative analysis in modern analytical chemistry and in pharmaceutical sciences. Clin Investig. 2024;14(9):524–35. doi: 10.20944/preprints202409.0057.v1

[11] Kaddah MMY, El Demellawy MA, Talaat W. Comprehensive analytical approaches: The use of LC-MS and IC-MS in modern pharmaceutical and biomedical sciences. Microchem J. 2025;216(May):114780. doi: 10.1016/j.microc.2025.114780

[12] Ng CYJ, Lai NPY, Ng WM, Siah KTH, Gan R-Y, Zhong LLD. Chemical structures, extraction and analysis technologies, and bioactivities of edible fungal polysaccharides from Poria cocos: An updated review. Int J Biol Macromol. 2024;261(Pt 1):129555. doi: 10.1016/j.ijbiomac.2024.129555 38278384

[13] Lai X, Kline JA, Wang M. Development, validation, and comparison of four methods to simultaneously quantify l-arginine, citrulline, and ornithine in human plasma using hydrophilic interaction liquid chromatography and electrospray tandem mass spectrometry. J Chromatogr B Analyt Technol Biomed Life Sci. 2015;1005:47–55. doi: 10.1016/j.jchromb.2015.10.001 26513134

[14] Wiśniewski J, Fleszar MG, Piechowicz J, Krzystek-Korpacka M, Chachaj A, Szuba A, et al. A novel mass spectrometry-based method for simultaneous determination of asymmetric and symmetric dimethylarginine, l-arginine and l-citrulline optimized for LC-MS-TOF and LC-MS/MS. Biomed Chromatogr. 2017;31(11):10.1002/bmc.3994. doi: 10.1002/bmc.3994 28436051

[15] Wu G, Meininger CJ. Analysis of citrulline, arginine, and methylarginines using high-performance liquid chromatography. Methods Enzymol. 2008;440:177–89. doi: 10.1016/S0076-6879(07)00810-5 18423217

[16] Gałęzowska G, Ratajczyk J, Wolska L. Determination of amino acids in human biological fluids by high-performance liquid chromatography: critical review. Amino Acids. 2021;53(7):993–1009. doi: 10.1007/s00726-021-03002-x 34028614 PMC8241665

[17] Wang HY, Hu P, Jiang J. Rapid determination of underivatized arginine, ornithine, citrulline and symmetric/asymmetric dimethylarginine in human plasma by LC-MS. Chromatographia. 2010;71(9–10):933–9. doi: 10.1365/s10337-010-1535-8

[18] Martens-Lobenhoffer J, Bode-Böger SM. Mass spectrometric quantification of L-arginine and its pathway related substances in biofluids: the road to maturity. J Chromatogr B Analyt Technol Biomed Life Sci. 2014;964:89–102. doi: 10.1016/j.jchromb.2013.10.030 24210895

[19] Li F, Li H, Jin X, Zhang Y, Kang X, Zhang Z, et al. Adipose-specific knockdown of Sirt1 results in obesity and insulin resistance by promoting exosomes release. Cell Cycle. 2019;18(17):2067–82. doi: 10.1080/15384101.2019.1638694 31296102 PMC6681786

[20] Leclercq IA, Da Silva Morais A, Schroyen B, Van Hul N, Geerts A. Insulin resistance in hepatocytes and sinusoidal liver cells: mechanisms and consequences. J Hepatol. 2007;47(1):142–56. doi: 10.1016/j.jhep.2007.04.002 17512085

[21] Søndergaard E, Jensen MD. Quantification of adipose tissue insulin sensitivity. J Investig Med. 2016;64(5):989–91. doi: 10.1136/jim-2016-000098 27073214

[22] Yang Y, Cruickshank-Quinn C, Armstrong M, Mahaffey S, Reisdorph R, Reisdorph N. Profiling of plasma results in improved coverage of metabolome. J Chromatogr A. 2014;217–26. doi: 10.1016/j.chroma.2013.04.030 PMC3734953 23672979

[23] Walker V, Mills GA. Quantitative methods for amino acid analysis in biological fluids. Ann Clin Biochem. 1995;32(1):28–57. doi: 10.1177/000456329503200103 7762950

[24] Moore SJ, MacDonald JT, Wienecke S, Ishwarbhai A, Tsipa A, Aw R, et al. Rapid acquisition and model-based analysis of cell-free transcription-translation reactions from nonmodel bacteria. Proc Natl Acad Sci U S A. 2018;115(19):E4340–9. doi: 10.1073/pnas.1715806115 29666238 PMC5948957

[25] Nijveldt RJ, Siroen MPC, van der Hoven B, Teerlink T, Prins HA, Girbes ARJ, et al. High plasma arginine concentrations in critically ill patients suffering from hepatic failure. Eur J Clin Nutr. 2004;58(4):587–93. doi: 10.1038/sj.ejcn.1601851 15042126

[26] Wu G, Bazer FW, Davis TA, Kim SW, Li P, Rhoads JM, et al. Arginine metabolism and nutrition in growth, health and disease. 2013;71(2):233–6. 10.1007/s00726-008-0210-y PMC2677116 19030957

[27] Marini JC, Agarwal U, Robinson JL, Yuan Y, Didelija IC, Stoll B, et al. The intestinal-renal axis for arginine synthesis is present and functional in the neonatal pig. Am J Physiol Endocrinol Metab. 2017;313(2):E233–42. doi: 10.1152/ajpendo.00055.2017 28611027 PMC5582884

[28] Morris SM Jr. Regulation of enzymes of the urea cycle and arginine metabolism. Annu Rev Nutr. 2002;22:87–105. doi: 10.1146/annurev.nutr.22.110801.140547 12055339

[29] Brosnan ME, Brosnan JT. Renal Arginine Metabolism. J Nutrition. 2004;134(10):2888–94. doi: 10.1093/jn/134.10.2791S 15465786

[30] Caldwell RW, Rodriguez PC, Toque HA, Narayanan SP, Caldwell RB. Arginase: A Multifaceted Enzyme Important in Health and Disease. Physiol Rev. 2018;98(2):641–65. doi: 10.1152/physrev.00037.2016 29412048 PMC5966718

[31] Tan B, Li X, Yin Y, Wu Z, Liu C, Tekwe CD, et al. Regulatory roles for L-arginine in reducing white adipose tissue. Front Biosci (Landmark Ed). 2012;17(6):2237–46. doi: 10.2741/4047 22652774 PMC3422877

[32] Bröer S, Bröer A. Amino acid homeostasis and signalling in mammalian cells and organisms. Biochem J. 2017;474(12):1935–63. doi: 10.1042/BCJ20160822 28546457 PMC5444488

[33] Marini JC, Didelija IC, Castillo L, Lee B. Glutamine: precursor or nitrogen donor for citrulline synthesis?. Am J Physiol Endocrinol Metab. 2010;299(1):E69-79. doi: 10.1152/ajpendo.00080.2010 20407005 PMC2904050

[34] Herman MA, She P, Peroni OD, Lynch CJ, Kahn BB. Adipose tissue branched chain amino acid (BCAA) metabolism modulates circulating BCAA levels. J Biol Chem. 2010;285(15):11348–56. doi: 10.1074/jbc.M109.075184 20093359 PMC2857013

[35] CARRUTHERS BM, WINEGRAD AI. Effects of insulin on amino acid and ribonucleic acid metabolism in rat adipose tissue. Am J Physiol. 1962;202:605–10. doi: 10.1152/ajplegacy.1962.202.4.605 13876898

[36] Arriarán S, Agnelli S, Remesar X, Alemany M, Fernández-López JA. White adipose tissue urea cycle activity is not affected by one-month treatment with a hyperlipidic diet in female rats. Food Funct. 2016;7(3):1554–63. doi: 10.1039/c5fo01503k 26901686

[37] Cao S, Gong W, Zhang X, Xu M, Wang Y, Xu Y, et al. Arginase promotes immune evasion of Echinococcus granulosus in mice. Parasit Vectors. 2020;13(1):49. doi: 10.1186/s13071-020-3919-4 32029006 PMC7006169

[38] Mori M. Regulation of nitric oxide synthesis and apoptosis by arginase and arginine recycling. J Nutr. 2007;137(6 Suppl 2):1616S-1620S. doi: 10.1093/jn/137.6.1616S 17513437

[39] Morris SM Jr. Recent advances in arginine metabolism: roles and regulation of the arginases. Br J Pharmacol. 2009;157(6):922–30. doi: 10.1111/j.1476-5381.2009.00278.x 19508396 PMC2737650

[40] Kurokawa T, An J, Tsunekawa K, Shimomura Y, Kazama S, Ishikawa N, et al. Effect of L-arginine supplement on liver regeneration after partial hepatectomy in rats. World J Surg Oncol. 2012;10:99. doi: 10.1186/1477-7819-10-99 22651848 PMC3449194

[41] Lecleire S, Hassan A, Marion-Letellier R, Antonietti M, Savoye G, Bôle-Feysot C, et al. Combined glutamine and arginine decrease proinflammatory cytokine production by biopsies from Crohn’s patients in association with changes in nuclear factor-kappaB and p38 mitogen-activated protein kinase pathways. J Nutr. 2008;138(12):2481–6. doi: 10.3945/jn.108.099127 19022976

[42] El-Sheikh NM, Khalil FA. L-arginine and L-glutamine as immunonutrients and modulating agents for oxidative stress and toxicity induced by sodium nitrite in rats. Food Chem Toxicol. 2011;49(4):758–62. doi: 10.1016/j.fct.2010.11.039 21130833

[43] Mendes-Ribeiro AC, Mann GE, de Meirelles LR, Moss MB, Matsuura C, Brunini TMC. The role of exercise on L-arginine nitric oxide pathway in chronic heart failure. Open Biochem J. 2009;3:55–65. doi: 10.2174/1874091X00903010055 19911071 PMC2775128

[44] Alvares TS, Conte CA, Paschoalin VMF, Silva JT, Meirelles C de M, Bhambhani YN, et al. Acute l-arginine supplementation increases muscle blood volume but not strength performance. Appl Physiol Nutr Metab. 2012;37(1):115–26. doi: 10.1139/h11-144 22251130

[45] Kanaley JA. Growth hormone, arginine and exercise. Curr Opin Clin Nutr Metab Care. 2008;11(1):50–4. doi: 10.1097/MCO.0b013e3282f2b0ad 18090659

