# Supplementary material for: Analysis of catabolic products of L-arginine; L-ornithine and L-citrulline and the residual L-arginine using the HPLC and LC-MS
Source: PLoS One. 2026 Apr 24;21(4):e0346976. doi: 10.1371/journal.pone.0346976 (PMC13108891; doi:10.1371/journal.pone.0346976)
Supplement: S1 File — (PDF) [file pone.0346976.s001.pdf]

Analysis of catabolic products of L-arginine; L-ornithine and L-citrulline and the residual L-arginine using the HPLC and LC-MS

Saranya Prashath¶

¶School of Biosciences, Division of Natural Sciences, University of Kent, UK.

Supporting information (SI)

Composition / formulation of complete DMEM and No L-Arg SILAC DMEM

The comparison of formulation of complete DMEM (Cat. No: 41966-029, Thermo Fisher) used in this study and formulation of L-arginine deficient media (Cat. No: 88364 Thermo Scientific) are presented in the below **table 1.** Thermo Fisher DMEM SILAC which is deficient in L-Arg and L-Lys, then L-lysine was backed in, and now the media is L-Arg deficient and used in this study. The composition of fetal bovine serum (non-USA Origin, Sigma, Cat. No: F7524) also detailed in **table 2**.

Table 1 Comparison of formulation of complete DMEM and no L-Arg DMEM SILAC media

S/N	Amino acids	Control Complete DMEM media		No L-Arg SILAC DMEM media	
		Concentration (mg/L)	µM	Concentration (mg/L)	µM
1	Glycine	30.0	400	30.0	399.6
2	L-Arginine HCl	84.0	398.1	L-Arg deficient	
3	L-Cystine 2HCl	63.0	201.3	63.0	201.3
4	L-Glutamine	580.0	3972.6	584.0	3995.9
5	L-Histidine HCl-H_2O	42.0	200	42.0	200.4
6	L-Isoleucine	105.0	801.5	105.0	801.5
7	L-Leucine	105.0	801.5	105.0	801.5
8	L-Lysine HCl	146.0	797.8	146.0 (added)	797.8
9	L-Methionine	30.0	201.3	30.0	201.1
10	L-Phenylalanine	66.0	400	66.0	399.5
11	L-Serine	42.0	400	42.0	399.7
12	L-Threonine	95.0	798.3	95.0	798.3
13	L-Tryptophan	16.0	78.4	16.0	78.3
14	L-Tyrosine	72.0	397.8	NF*	NF*
15	L-Tyrosine-2NA-2H_2O	NF*	NF*	104.0	398.8
16	L-Valine	94.0	803.4	94.0	803.4

*NF-Not Found

Table 2 The composition of fetal bovine serum

Test	Specification	Result
Protein content	30 - 45 g/L	34.8 g/L
Albumin	Report result	15.8 g/L
Alpha globulins	Report result	16.7 g/L
Beta globulins	Report result	2.1 g/L
Gamma globulins	Report result	0.3 g/L
Igg (Immunoglobulin g)	Report result	164.8 mg/L

In this study we used two different culture media (complete DMEM and No L-Arg SILAC DMEM) that consisted of different concentrations of amino acids (**Table 3**) and FBS (10% v/v). FBS itself contains protein and amino acids (**Table 2**) to facilitate the growth of the cells. Therefore, baseline content of selected amino acids in the control complete DMEM media with 10% FBS and no L-Arg SILAC DMEM media with 10% FBS was analysed in three biological replicates by HPLC (**Table 3)**. The HPLC data confirmed that the concentration of L-Arg in no L-Arg was 0 µM and the concentration of L-Arg in the control complete DMEM was 250.26 µM.

Table 3 Selected amino acids concentration in growth media with 10 % FBS

**Samples**	**Concentration**		
	**L-Arginine (µM)**	**L-Citrulline (µM)**	**L-Ornithine (µM)**
Control complete DMEM media with 10%FBS	250.256	45.213	11.909
No L-Arg SILAC DMEM media with 10%FBS	0.000	55.712	14.978

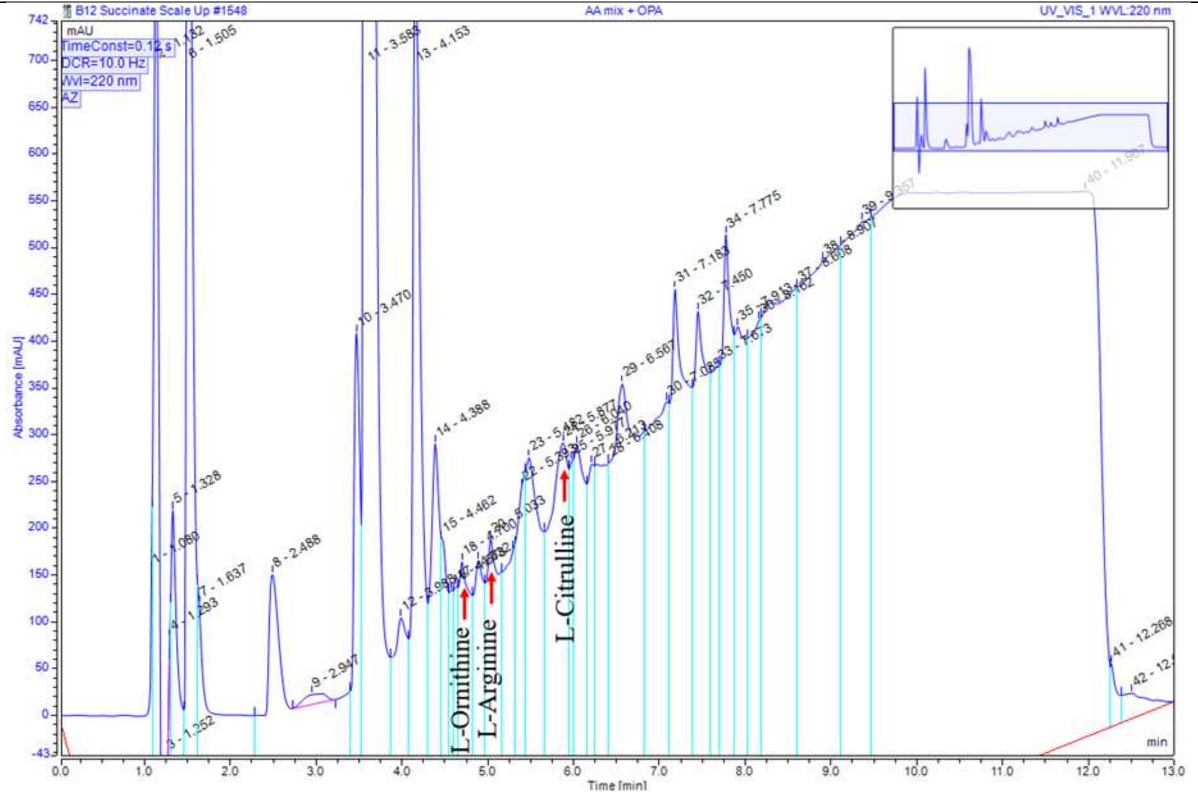

Fig A Liquid chromatography trace showing components present in a sample consisting of the standard amino acids mix after derivatization with OPA, and the retention time (min) and absorbance (mAU) of each peak. Mass spectra of the target amino acid adducts labelled in this LC-chromatogram are shown in the figures below.
