# Supplementary material for: Analysis of catabolic products of L-arginine; L-ornithine and L-citrulline and the residual L-arginine using the HPLC and LC-MS
Source: PLoS One. 2026 Apr 24;21(4):e0346976. doi: 10.1371/journal.pone.0346976 (PMC13108891; doi:10.1371/journal.pone.0346976)
Supplement: S2 File — (PDF) [file pone.0346976.s002.pdf]

Analysis of catabolic products of L-arginine; L-ornithine and L-citrulline and the residual L-arginine using the HPLC and LC-MS

Saranya Prashath[¶]

[¶]School of Biosciences, Division of Natural Sciences, University of Kent, UK.

Statistical Tables

HPLC analysis of residue L-arginine, and L-ornithine and L-citrulline in the cell culture media of BNL CL2 cells cultured in different initial L-arginine concentrations by HPLC

Table 1 Statistical table for the effect of exogenous L-arginine concentration on quantification of residual serum L-Arg.

Source of Variation	P value	P value summary	Significant?
**Interaction**	<0.0001	****	Yes
**Time**	<0.0001	****	Yes
**L-Arg +/- and Control (Com)**	<0.0001	****	Yes

Table 2 Statistical table for the effect of exogenous L-arginine concentration on quantification of residual serum L-Cit.

Source of Variation	P value	P value summary	Significant?
**Interaction**	<0.0001	****	Yes
**Time**	<0.0001	****	Yes
**L-Arg +/- and Control (Com)**	<0.0001	****	Yes

Table 3 Statistical table for the effect of exogenous L-arginine concentration on quantification of residual serum L-Orn.

Source of Variation	P value	P value summary	Significant?
**Interaction**	<0.0001	****	Yes
**Time**	<0.0001	****	Yes
**L-Arg +/- and Control (Com)**	<0.0001	****	Yes

Table 4 Samples comparison for the effect of exogenous L-arginine concentration on quantification of residual serum L-Arg, L-Cit and L-Orn.

Tukey's multiple comparisons test	Amount of L-Arg			Amount of L-Cit			Amount of L-Orn		
	T=0	24 h	72 h	T=0	24 h	72 h	T=0	24 h	72 h
Untreated vs. Cont. Com	****	****	****	****	ns	ns	**	**	****
Untreated vs. 400 µM	****	****	****	****	ns	ns	**	**	****
Untreated vs. 800 µM	****	****	****	****	ns	ns	**	***	****
Untreated vs. No L-Arg	****	****	****	****	ns	ns	**	*	****
Cont. Com vs. 400 µM	ns	ns	ns	ns	ns	ns	ns	ns	*
Cont. Com vs. 800 µM	ns	ns	****	ns	ns	ns	ns	ns	ns
Cont. Com vs. No L-Arg	ns	*	****	ns	ns	ns	ns	ns	ns
400 µM vs. 800 µM	ns	ns	****	ns	ns	ns	ns	ns	ns
400 µM vs. No L-Arg	ns	ns	****	ns	ns	ns	ns	ns	ns
800 µM vs. No L-Arg	ns	**	*	ns	ns	ns	ns	ns	ns

HPLC analysis of residue L-arginine, and L-ornithine and L-citrulline in the cell culture media of 3T3 L1 cells cultured in different initial L-arginine concentrations by HPLC

Table 5 Statistical table for the effect of exogenous L-arginine concentration on quantification of residual serum L-Arg.

Source of Variation	P value	P value summary	Significant?
**Interaction**	<0.0001	****	Yes
**Time**	<0.0001	****	Yes
**L-Arg +/- and Control (Com)**	<0.0001	****	Yes

Table 6 Statistical table for the effect of exogenous L-citrulline concentration on quantification of residual serum L-Cit.

Source of Variation	P value	P value summary	Significant?
**Interaction**	<0.0001	****	Yes
**Time**	<0.0001	****	Yes
**L-Arg +/- and Control (Com)**	<0.0001	****	Yes

Table 7 Statistical table for the effect of exogenous L-ornithine concentration on quantification of residual serum L-Orn.

Source of Variation	P value	P value summary	Significant?
**Interaction**	<0.0001	****	Yes
**Time**	<0.0001	****	Yes
**L-Arg +/- and Control (Com)**	<0.0001	****	Yes

Table 8 Samples comparison for the effect of exogenous L-arginine concentration on quantification of residual serum L-Arg, L-Cit and L-Orn.

Tukey's multiple comparisons test	Amount of L-Arg			Amount of L-Cit			Amount of L-Orn		
	T=0	24 h	72 h	T=0	24 h	72 h	T=0	24 h	72 h
Untreated vs. Cont. Com	****	ns	****	****	ns	ns	**	***	****
Untreated vs. 400 µM	****	****	****	****	ns	ns	**	****	****
Untreated vs. 800 µM	****	****	****	****	ns	ns	**	****	****
Untreated vs. No L-Arg	****	****	ns	****	ns	ns	**	****	**
Cont. Com vs. 400 µM	ns	****	*	ns	ns	ns	ns	**	****
Cont. Com vs. 800 µM	ns	****	****	ns	ns	ns	ns	***	****
Cont. Com vs. No L-Arg	ns	****	****	ns	ns	ns	ns	****	****
400 µM vs. 800 µM	ns	****	****	ns	ns	ns	ns	ns	ns
400 µM vs. No L-Arg	ns	ns	****	ns	ns	ns	ns	***	*
800 µM vs. No L-Arg	ns	****	****	ns	ns	ns	ns	*	ns
